# Endometriosis: A New Cellular and Molecular Genetic Approach for Understanding the Pathogenesis and Evolutivity

**DOI:** 10.3389/fsurg.2014.00016

**Published:** 2014-05-27

**Authors:** Jean Bouquet De Jolinière, Jean Marc Bernard Ayoubi, Luca Gianaroli, Jean Bernard Dubuisson, Jean Gogusev, Anis Feki

**Affiliations:** ^1^Maternity and Surgical Department of Gynecology, HFR Hôpital Cantonal Fribourg, Fribourg, Switzerland; ^2^Endodiag Research Laboratory Genopole, Evry, France; ^3^Department of Gynecologic Surgery, Foch Hospital, Suresnes, France; ^4^S.I.S.Me.R. Reproductive Medicine Unit, Bologna, Italy; ^5^INSERM U507, Hospital Necker, Université Paris Descartes, Paris, France

**Keywords:** endometriosis, permanent cell line, chromosome, comparative genomic hybridization, fluorescence *in situ* hybridization, embryonic duct remnants

## Abstract

Endometriosis is a benign disease with high prevalence in women of reproductive age estimated between 10 and 15% and is associated with considerable morbidity. Its etiology and pathogenesis are controversial but it is believed to involve multiple genetic, environmental, immunological, angiogenic, and endocrine processes. Altered expressions of growth factors, cytokines, adhesion molecules, matrix metalloproteinases, and enzymes for estrogen synthesis and metabolism have been frequently observed in this condition. The possibility of genetic basis of endometriosis is demonstrated in studies of familial disease, in which the incidence of endometriosis is higher for first-degree relatives of probands as compared to controls. This review describes mainly the cellular, cytochemical, cytogenetic, and molecular genetic features of endometriotic lesions and cultured endometriotic cells. In attempts to identify candidate gene (s) involved in the pathogenesis of endometriosis, a tissue-based approaches including conventional cytogenetics (RHG-banding), loss of heterozygosity (LOH), and comparative genomic hybridization (CGH) were employed. In addition to the karyotypic anomalies, consistent chromosome instability was confirmed by CGH and fluorescence *in situ* hybridization (FISH). The nature and significance of the molecular genetic aberrations in relation to the locations and function of oncogenes and tumor suppressor genes will be discussed. At last, a possible pathogenic role of embryonic duct remnants was observed in seven female fetal reproductive tract in endometriosis and may induce a discussion about the beginning of ovarian tumors and malignant proliferations.

## Introduction

Endometriosis is a common gynecological disorder accounting for 10–15% of pelvic pain and infertility in women of reproductive age. Despite its prevalence, relatively little is known about the underlying pathophysiological mechanisms. Numerous studies however suggest that it has a multidimensional etiology including, hereditary, hormonal, and immunological factors (1, 2).

This condition is considered as benign disorder, but exhibits cellular proliferation, cellular invasion, and neo angiogenesis. The glandular epithelium occasionally exhibits cytological atypia and/or hyperplasia (3) as well as DNA aneuploidy (4). The malignant potential of the endometriotic epithelial cells has been suggested in some cases considering the monoclonal methylation pattern of the HUMARA alleles and their invasive and metastatic ability *in vitro* (5, 6). In this context, previous studies supported the hypothesis that progression of endometriotic lesions to frank malignancy can occur although with a very rare incidence (7–11). However, the significance of the so-called malignant potential of endometriotic cells is controversial since monoclonal cells growth pattern was described in various benign lesions (5).

It is difficult to obtain direct evidence for mechanistic events in endometriosis for a variety of reasons. One is a lack of experimental models that adequately mimic *in situ* conditions. Animal models of endometriosis, exist including monkey and mouse systems (12, 13), but at present it is not known how closely the disease in animals mimics the human disease. More information critical to the understanding of pathogenesis and treatment of endometriosis will possibly come from studies of controlled *in vitro* cell models. Cell culture allows the study of cell specific characteristics of endometriotic tissues compared to epithelial and stromal cells from normal endometrium. Such cellular systems will permit the investigation of regulatory mechanisms controlling steroid hormone receptors expression and cytokine and growth factor secretion, which have been postulated to differ between cells from normal endometrium and endometriotic lesions (14–18).

## Endometriotic Cell Growth and Differentiation

There is strong evidence that the endometrium of women with endometriosis has an increased capacity to proliferate, implant, and grow in the peritoneal cavity (2). Morphological studies of tissue sections have demonstrated that the endometriotic glands have a wide range of morphologic development presenting a pattern ranging from poorly to highly differentiated glandular structures. Such morphological variations occur from gland to gland even within cellular areas in the same gland (19). Adequate morphological changes of the endometriotic glands were found in implants only during the proliferative phase of the menstrual cycle, whereas secretory changes were completely missing during the luteal phase (19). These changes include histological differentiation and induction of secretory proteins such as prolactin (PRL) (20), insulin-like growth factor binding protein-1 (IGF-BP1) (21), and extracellular matrix proteins such as fibronectin (22). In addition, several markers are aberrantly expressed in endometriotic cells in comparison to the normal cell elements, including the gap junction connexins (Cx) (23), β-cadherins (24), metalloproteinases (25), and P450 aromatase (26). Altogether, these features indicate a high degree of dedifferentiation in comparison to the normal situation.

The regulatory mechanisms involved in morphological and biochemical differentiation of uterine endometrium are obviously complex (27, 28). It is widely accepted that endometrial stromal cells are essential for proliferation, morphogenesis, and differentiation of epithelial cells. Current models of how signaling may be accomplished between stromal and epithelium include transmission via diffusible substances, via the extracellular matrix, and via direct cell–cell contact. Growth factors and organ specific paracrine factors are potential candidates produced by the stromal cells that affect the endometrial epithelium (29, 30). In this respect, it has been reported that endometrial stromal cells from women with endometriosis secrete a greater amount of IGF-I and -II (31) and hepatocyte growth factor (32). Signaling through direct stromal–epithelial contact may also be accomplished via interactions between complementary cell surface adhesion molecules (33, 34).

The dynamics of the differentiation process for human endometriotic proliferations can be studied only in cell culture. Such approach should provide information about this process *in vivo*, particularly about how changing protein synthesis accompanies changing cell structure. To date, little is known about mechanisms controlling endometriotic cell differentiation *in vitro*. Primary endometriotic cell cultures retain some aspects of differentiation as a manifest of appropriate protein synthesis. Comparatively, eutopic endometrial cells induced to differentiate produce domes, gland-like structures, polarized sheets, and spheroids (35). Conversely, cultures of stromal-like cells derived from endometriotic tissues display a variety in morphology indistinguishable from normal endometrial stroma, containing both fibroblast-like cells and cells with more rounded shape (36). However, experimental work suggests that endometriotic cells from peritoneal implants have an innate proliferative abnormality compared to eutopic cells in that they undergo more rounds of cell division and can be maintained in a stationary but functional state for longer periods of time in primary culture.

## Endometriosis in Tissue Culture: Phenotype and Growth Characteristics

Ectopic endometrial cells have been poorly investigated, mainly due to the rare availability of endometriotic tissue required for cell culture and the limited number of cells, particularly those of epithelial phenotype. Long term culture of endometriotic cells appears to be a reliable method to study the proliferative characteristics, the cellular capacity for differentiation, and the nature of the biologic products released.

So far, establishment of human endometriosis derived permanent cell lines were exceptionally successful. Two recently described *in vitro* models, including the FbEM-1 cell line (37) and cell lines obtained after immortalization of endometriotic cells with simian virus 40 (38), may represent a valuable material for further studies of endometriotic cell growth and differentiation. However, the proposed *in vitro* cell models have their limitations since endometriotic lesions are histologically complex containing both glandular and stromal elements. Thus, immortalized cell lines with one cell type, usually exhibiting features of undifferentiated cells, do not accurately represent the *in vivo* situation.

In an effort to learn more about endometriotic cell growth and differentiation, a cell culture model from different types of endometriotic lesions including ovarian cysts, peritoneal implants, and deep infiltrating endometriosis was successfully established in the author’s laboratory. Endometriosis cultures are in general prepared from biopsies from the various lesions as essentially described (37) with some modifications.

Briefly, the fragments obtained at surgery are rinsed, minced, and digested with collagenase (2 mg/ml) at 37°C in 5% CO_2_ during 120 min. Epithelial glands are separated from stromal cells, blood cells, and debris by serial filtration using narrow gage sieves (45 μm). Thereafter, the glands are washed from the sieves and allowed to proliferate in Dulbecco’s modified Eagle medium supplemented with 10% fetal calf serum and antibiotics. In separate experiments, stromal cells from the collagenase digested endometriotic tissues are allowed to adhere to the culture flasks and maintained *in vitro* following the same culture procedures. The primary cultures obtained from the peritoneal biopsies and from the chocolate cyst wall contain mostly mixed cell populations variable in their size and in their morphology. Within 1 week, small foci consisting of rather small spindle or polygonal cells appear among the loosely proliferating epithelial-like cells and gradually increase in size. After 3–4 weeks in culture, the cellular monolayer is successfully passaged *in vitro* at a density of 2 × 10^5^ cells/ml.

**Figure 1:**
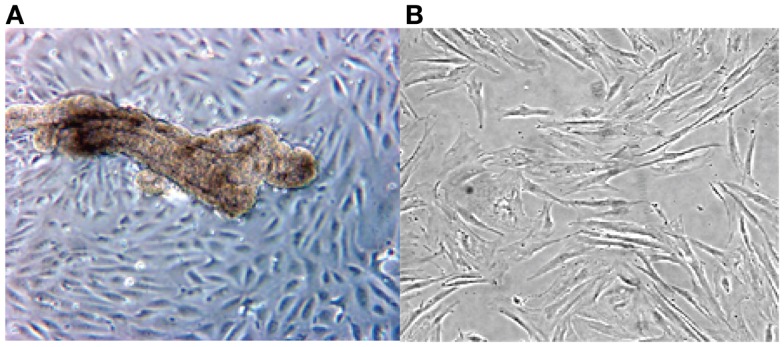
**Phase contrast microscopy of a primary culture of a fragment from ovarian endometrioma (A)**. Note presence of an endometriotic gland giving rise to a monolayer of epithelial-like cells **(A)**. In **(B)**, aspect of elongated fibroblast-like cultured cells obtained from the stromal component of a large peritoneal endometriotic implant **(B)**.

The cultured stromal cells from both endometriotic implants and the endometrioma wall appear as adherent fibroblast-like cells, their doubling time being approximately 9 days. These stromal elements usually proliferate as elongated cells, densely packed, and reach confluence in about 10–14 days. An aspect on phase contrast microscopy of an endometriotic gland from a peritoneal implant in primary culture giving rise to growing epithelial-like cells and third passage of culture derived from an ovarian endometrioma is presented in Figure 1.

To determine if the cultured endometriotic cells retain specific epithelial markers, immuno-cytochemistry is routinely performed using a panel of specific antibodies. For immunostaining, the endometriotic cells cultured either in tissue culture chamber slides (Labtek) or prepared as cytocentrifuge smears are fixed in 4% paraformaldehyde or in cold acetone (10%), rinsed in PBS, and incubated with the primary antibody. Monoclonal and/or polyclonal antibodies are revealed using rabbit anti-mouse or goat anti-rabbit IgG peroxydase conjugated antibodies (Dako, Denmark) according to standard methods. Control slides are incubated with normal mouse serum to replace the specific monoclonal or polyclonal antibodies.

The results of the immuno-histochemical staining of both epithelial and stromal endometriotic cells with specific monoclonal and polyclonal antibodies are summarized in Table 1.

**Table 1:** **Phenotype of endometriotic cell cultures**.

Antibody used	Specificity	Source^a^	Epithelial cells^b^	Stromal cells^b^
CK (5D3)	Cytokeratins 8, 18, 19	Boehringer	60	15
V9	Vimentin	Dako	30	75
E29	EMA	Dako	–	–
Factor VIII	vWF	Dako	–	–
IOT2	HLA cl.I	Immunotech	40	35
CD3 (UCHT1)	T cells	Dako	–	–
CD20 (B-Ly)	B cells	Dako	–	–
CD45 RB	T, B, Mo, Mf, Gr	Dako	–	–
R (B-30)	Progesterone receptor	Santa Cruz	40	21
AR (C-19)	Androgen receptor	Santa Cruz	<10	10
ER (H-20)	Oestrogen receptor	Santa Cruz	–	24
DE-R11	Desmin	Dako	–	2
CIV22	Collagen IV	Dako	–	–
CEA (II-7)	Carcinoembryonic antigen	Dako	–	–
OC-125	CA-125	Dako	–	–

*CA-125 = carcinoma antigen-125; EMA = epithelial membrane antigen; vWF = von Willebrandt factor; HLA = human leukocyte antigen*.

*^a,b^ Percentage of positive epithelial and stromal cells*.

**Figure 2:**
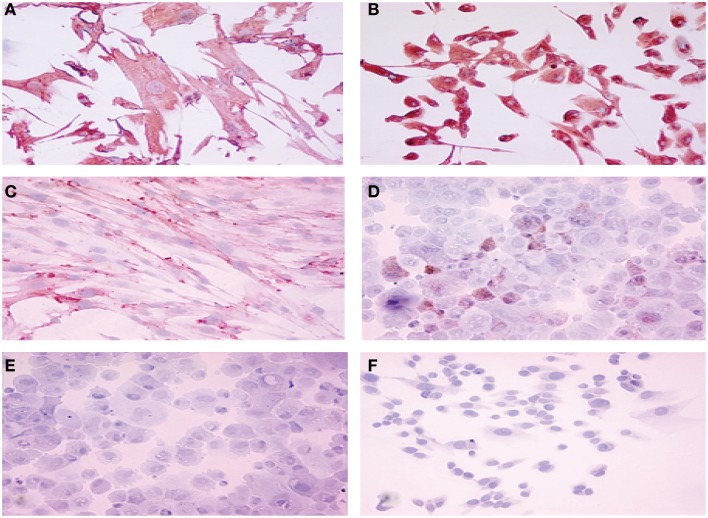
**Immuno-cytochemical localization of cytokeratin (A), vimentin (B), estrogen receptor (C), progesterone receptor (D), androgen receptor (E) and factor VIII (F) proteins in cultured endometriotic cells (second passage)**. The culture was established from a large peritoneal implant (black lesion). Note presence of large polymorphic adherent cells growing in monolayer. Original magnification ×400; in B,C,D,E,F (× 280).

Among the epithelial markers, cytokeratin expression remained one of the most specific characteristics of the cultured cells. The anti-cytokeratin antibody detecting cytokeratin 8, 18, and 19 produce cytoplasmic immunolabeling of the majority of epithelial endometriotic cells from various passages (Figure 2). These cells were negative for factor VIII-related antigen, indicating no contamination with endothelial cells. More than 40% of the cells were immunoreactive with the anti progesterone receptor (PR) antibody showing brown nuclear stain produced by the diaminobenzidine (DAB) colorimetric reaction (Figure 2). Conversely, less then 15% were stained with anti androgen receptor antibody while only weak immunostaining was obtained for estrogen receptor (2–5%). For comparison, both cytokeratins and vimentin were expressed in endometriotic stromal cells. Thus, most of the phenotypic features of the normal endometrium are retained in the cultured endometriotic cells (Figure 2).

**Figure 3:**
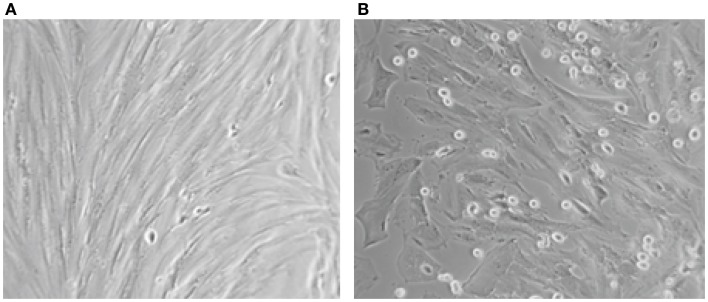
**Aspect on phase contrast microscopy of untreated (A) and endometriotic cells treated with 2-Ethoxyestradiol (1 mM) during 24 h (B)**. A striking morphological change was obtained after 12 h of treatment. The 2-Ethoxyestradiol induced decrease of DNA synthesis by 68% after 48 h of treatment (BrDU incorporation).

Concerning the differentiation capacity, experiments were performed to determine whether the cultured epithelial and stromal endometriotic cells undergo changes after treatment with the estradiol metabolite 2-Ethoxyestradiol and medroxyprogesterone acetate (MPA). Phase contrast microscopy indicated that cells cultured in the absence of steroids remained growing until confluence. For cultures grown in medium supplemented with 1% of FCS, addition of with 2-Ethoxystradiol (1 μM) resulted in strong inhibition of cell growth and appearance of morphological changes, i.e., the cells became large with appearance of cytoplasmic projections (Figure 3). In contrast, addition of MPA (1 μM) resulted in an approximately twofold stimulation of the cell growth. When cultured in Matrigel for 96 h, the number of MPA-treated cells was considerably greater than that of untreated cells. Occasionally, some of the cells exhibit morphological changes and develop a polygonal appearance (not shown). This phenomenon may be explained by the increased level of adhesion molecules in MPA-treated endometriotic cells promoting the aggregation (33, 34).

## Cytogenetic and Molecular Genetic Analysis of Endometriosis

Most genetic changes occur in somatic cells, but some occur as germ line defects that can result in an inherited predisposition to endometriotic development. In this context, convincing evidence in favor of a genetic basis for endometriosis is provided by several studies sowing that the prevalence of the disease is higher in the first-degree relatives of affected women than in controls thought to be representative of the general population (39–43).

At the tissue level, cytogenetic, molecular genetic, and molecular cytogenetic techniques have been applied to endometriosis and have lead to the identification of consistent somatic genetic alterations. Altogether, these findings strongly suggest existence of genomic aberrations in endometriotic tissues probably related to distinct putative genes involved in development of the disease.

### Chromosomal anomalies observed in endometriotic cells

To date, conventional cytogenetic studies have been poorly informative because of the rare availability of endometriotic tissue required for cell culture and the limited yield of endometriotic cells. No specific chromosomal abnormalities have been identified by conventional cytogenetic analysis applied to date, to a total of 73 published cases of endometriotic lesions (44, 45). The limited information yielded by conventional karyotypic studies reflects two factors; the difficulties specifically related to endometriotic tissues and those inherent to the technique. In fact, culture of pure endometriotic cells is hampered by the mixture of epithelial and stromal cells in addition to inflammatory infiltrate containing fibroblasts and histiocytic cells. Alternatively, the predominantly normal karyotype seen in endometriotic lesions may be due to resolution limits of karyotyping, with any changes present being submicroscopic. Moreover, there may be overgrowth of normal cells or selection *in vitro* against aneuploid cells. The evidence for the latter comes from a study where the FISH was performed on direct preparation revealing clonal aberrations and aneuploidy of several chromosomes (46). This selection against aneuploid cells may be a result of the methods used in preparation of the tissue. It is noteworthy that collagenase, used to disrupt the lesion prior to culture, results in loss of the aneuploid population that is not seen when mechanical disruption alone is used. Despite the difficulties of cytogenetic analysis discussed above, it nevertheless has an important role to play in understanding of endometriosis. It is important to emphasize, for example, that cytogenetic analysis is the only technique among those reviewed here that has the ability to identify novel chromosomal translocations.

**Figure 4:**
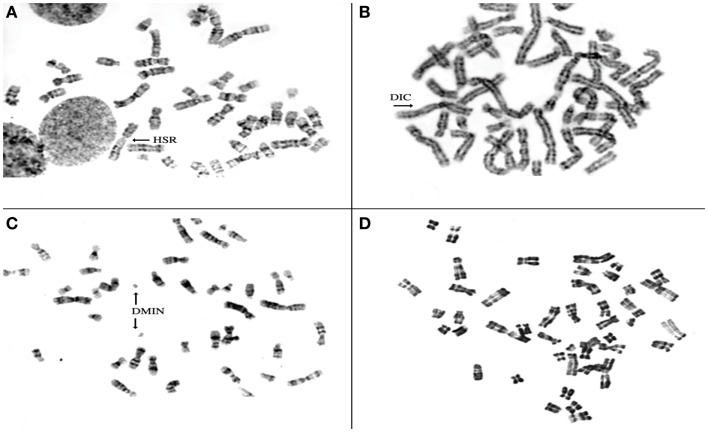
**Representative metaphase spreads obtained from four different endometriotic lesions**. Note presence of structural chromosomal alterations expressed as aneuploid metaphase with homogeneous staining region (HSR) in **(A)**, dicentric chromosomes (DICs) **(B)**, double minute chromosomes D-MIN **(C)**, premature centromeric disjunctions **(D)**.

To investigate the chromosomal structure of the different types of endometriotic lesions, we performed conventional karyotype analysis of a series of seven endometriotic samples obtained from patients with advanced stage disease. In strictly defined culture conditions, increased percentage of aberrant metaphases showing aneuploidy, dicentric chromosomes, endomitoses, and chromosomal pulverization were detected in five of seven studied samples by RHG-banding (Figure 4). These results were further extended by multicolor fluorescence *in situ* hybridization analysis (FISH) with painting probes for chromosomes 1, 6, 7, 8, 11, 12, 17, and 22.

FISH analysis with painting probe for chromosome 17, demonstrated presence of three copies of that chromosome in 17% of the analyzed metaphases in one ovarian endometrioma. In contrast, loss of chromosome 17 was detected in 2 cases of 7 studied. Loss of the q arm of chromosome 22 was detected in 3 cases (not shown). Similar data were previously reported by Shin et al. (46) in one case of four endometriotic samples studied. Thus, it could be hypothesized that at least chromosomes 1, 16, 17, and 22 exhibit structural aberrations containing genes that might play a role in the development and/or progression of endometriosis. In this context, it should be underlined that endometriotic tissue sampling and culture conditions are of a major importance in successful detection of aberrant metaphases.

#### Loss of heterozygosity

Only a limited number of loss of heterozygosity (LOH) studies have been conducted using DNA from histologically homogeneous endometriotic tissues. Recent molecular allelotyping work has detected somatic genetic changes, distributed along several chromosomes including chromosome 9p, 11q and 22q in 11 of 40 (28%) examined endometriotic samples (47). When more informative loci markers were applied, allelic imbalances were revealed in 82% of the endometriotic lesions diagnosed simultaneously with ovarian carcinoma (48). However, allelotyping studies are limited to analyzing specific regions of the genome for example detecting loss of part of a chromosome arm in a set of lesions. They are also limited by the need to have endometriotic tissue with minimal normal contaminating tissue and normal tissue from the same patient to serve as a control. Nevertheless, these studies were remarkable enough to point out that tumor suppressor gene (s) inactivation may play a role in the development of endometriosis.

#### Fluorescence *in situ* hybridization

In general, FISH analysis has revealed more clonal aberrations than conventional cytogenetic analysis in a number of altered tissues. This approach has the advantage that culturing of endometriotic cells is unnecessary and eliminates the problems of tissues heterogeneity. Performed on interphase nuclei in severe/advanced stage endometriosis, higher frequency of monosomy for chromosome 16 and 17 and increased number of cells with trisomy 11 were recently reported (46, 49). The limitations of studies using FISH strategy is that only centromeric probes have been used and not all chromosomes have been investigated. The information gained using centromeric probes is extrapolated to the whole chromosome, an assumption which may not be accurate.

### Genetic aberrations in endometriosis detected by comparative genomic hybridization

Comparative genomic hybridization (CGH) is a molecular cytogenetic method able to discover and map genomic regions for chromosomal gains and/or losses in a single experiment (50). Regions showing an increased copy number (gain or amplification) may harbor dominant oncogenes, whereas regions with a decreased copy number (loss) may contain tumor suppressor genes. In this regard, the CGH approach has been successfully applied in studies of solid tumors including endometrial (51) and ovarian carcinomas (52), and ovarian carcinoma cell lines (53).

**Figure 5:**
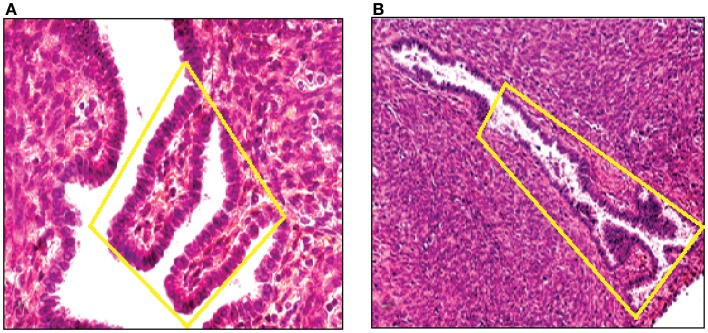
**DNA was extracted from a tissue section of a large peritoneal endometriotic implant with presence of an endometriotic gland containing papillary projections [Hematoxylin Eozin stained (A), and from a gland of tissue section from rectovaginal endometriotic nodule (B)]**.

By means of CGH, we screened primary endometriotic lesions for chromosomal gains and/or losses in a series of patients with advanced stage disease. The study was performed on native, non-amplified DNA extracted from manually dissected endometriotic tissues (Figure 5). Recurrent gene copy number alterations were found in 15 of 18 (83%) of cases with advanced stage endometriotic lesion indicating clonal genetic changes. Losses which predominated over gains showed clustering at certain chromosomal regions suggesting a recurrent non-random pattern of chromosomal alterations. Imbalances were not found in three cases, yet existence of balanced reciprocal translocations, very small chromosomal imbalances or mutations can not be excluded since such alterations are not detectable by CGH.

The average number of copy alterations in our series of endometriotic tissues was 3.1 per lesion, which is low compared to malignant tissues (52). The DNA copy number abnormalities were not related to the histological type or to the site of the endometriotic lesion. Remarkably, low frequencies of somatic genetic changes by CGH analysis were demonstrated in other benign proliferations (54, 55).

**Figure 6:**
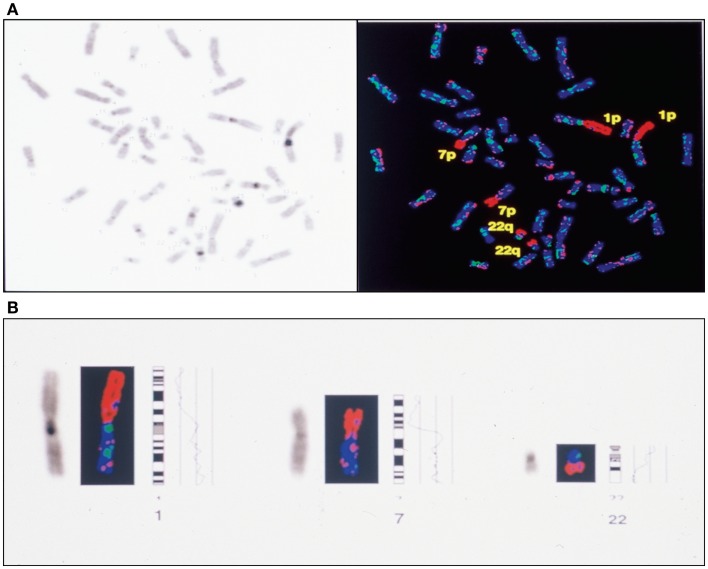
**Complete CGH analysis of one large endometriotic peritoneal implant presented in (A)**. Compilation of fluorescence ratio profiles from 10 different metaphases was used to calculate the average ratio profiles of this sample. As shown, copy number losses are distributed along chromosomes 1p, 7p and 22q. DAPI staining on the left, and digitized fluorescent image on the right. More detailed representation of the deleted chromosomal segments located on chromosomes 1, 7 and 22 **(B)**.

The most common regions of loss of genomic material were located on 1p involving at least the 1p32–36 (50%), 5p (33%), 6q (27%), 7p14-p22ter (22%), 16qter (22%), and 22q12.3-qter (50%) segments (Table 2; Figure 6). The other less common copy number changes included loss involving chromosome arms 9q (22%), 16q (22%), and 17q, found in one case. Underrepresentation of 1p was recurrently found in 9 of 18 studied cases. DNA sequences losses were restricted to the 1p36 band in three cases; the remaining six were larger deletions. Deletions of chromosome 1 were particularly common in all types and stages of endometriotic tissues including the peritoneal implants, endometriomas, and umbilical nodules. Gains were less commonly found and were localized on chromosomes 6q and 17q in 2 cases (Table 2).

**Table 2:** **Genetic aberrations in endometriotic tissues detected by comparative genomic hybridization**.

Case (*)	Stage	Chromosome imbalances
1	IV	dim (1p, 7p, 22q)
2	IVa	dim (5p12-ter, 16q, 17qter, 22q)
3	IV	dim (1p36-ter, 16q, 22q)
4	III	dim (1p, 6q, 7p, 21q, 22q)
5	IVa	enh (7q)
6	IV	None
7	III	None
8	IVa	dim (1p36-ter, 6q, 9qter, 19q)
9	IVa	dim (7)
10	IVa	dim (1p, 5p, 8p, 11p, 16q, 17p)
11	IVa	enh (1q), dim (5p, 9q, 16q)
12	IVb	dim (1p36-ter, 2p, 5p, 6q, 7q, 11q)
13	IVa	dim (3q, 5p, 6q, 9q, 22q)
14	IV	dim (1p, 7p, 22q)
15	IVb	enh (6q), dim (2p21,5p,7p, 9q, 12q,19q,22q)
16	IV	None
17	IV	dim (1p, 6q, 22q)
18	IV	enh (17q), dim (1p, 22q)

*Large peritoneal implants (case 1–6); umbilical endometriotic nodules (case 7–8); ovarian endometriomas (case 9–18).

Deletions of the short arm of chromosome 1 are of particular interest since they were found in 50% of the cases with advanced stage endometriosis. Chromosome 1p is known to harbor putative tumor suppressor genes associated with a variety of tumors including neuroblastomas, pheochromocytomas, melanomas, and carcinomas of the liver, breast, and colon (56) although, to date, none of these gene (s) has been cloned. They include ID3, CDC2L1, DAN, PAX7, E2F2, TNFR2, and TCEB3 (57).

Other relevant copy number losses indicative of chromosomes where tumor suppressor genes reside were mapped to chromosomes 5p, 6q, and 22q. Copy number changes on both chromosomes 5p and 6q were detected in approximately one third of the endometriotic cases studied. Previous allelotyping studies have reported high percentage of LOH using markers located at 5p and 6q chromosomal segments in numerous human malignancies. For example, consistent copy number losses on 5p were detected in papillary thyroid cancer (58), human male germ tumors (59), and in childhood acute lymphoblastic leukemia (60). Concerning the chromosome 6 it has been suggested that a putative tumor suppressor gene located on the 6q arm is involved in development of ovarian carcinoma (61). A potential candidate tumor suppressor gene for this region may be the superoxide dismutase gene 2 (SOD2) gene located on 6q25. High frequencies of LOH encompassing the SOD2 gene have been shown in ovarian carcinoma (61).

Another region found recurrently deleted in two endometriotic peritoneal implants and two large ovarian endometriomas is located on the long arm of chromosome 16. Reduced copy number on 16q may facilitate inactivation of the cell adhesion molecule E-cadherin (62) and the cell adhesion regulator CAR gene product (63). Loss of DNA sequences on 16q could also be related to the decreased expression of E and N-cadherin molecules recently reported in a series of endometriomas, cystadenomas, borderline, and carcinomas of the ovary (64).

A striking finding is the underrepresentation of chromosome 22 seen in 50% of the cases analyzed. DNA copy number loss interpreted as monosomy of chromosome 22 has been described notably in various neoplasms, including ovarian carcinoma (65), meningioma (66), and mesothelioma (67). One candidate for the tumor suppressor gene in chromosome 22q is neurofibromatosis type 2 (NF2) gene at 22q12 (68).

The molecular cytogenetic data identified by CGH in primary endometriotic lesions correspond in part to previous molecular allelotyping findings (47, 48). Our results highlight several novel regions located on chromosomes 1p, 6q, and 22q that might harbor single or multiple tumor suppressor genes involved in pathogenesis of endometriosis. Altogether, the non-random distribution coupled with the subchromosomal localization of the genetic alterations strongly supports the idea that these abnormalities are relevant to and associated with the endometriotic process.

## Occurrence of chromosomal instability in edometriotic lesions

It is defined as an alteration of the chromosome constitution occurring in various pathological conditions:

– Fundamental property of neoplastic cells (most malignant and benign tumors) (69).
– Precancerous lesions (dysplasia, leucoplasia, and cystically altered tissues).
– Chronic inflammatory conditions.
– Infectious diseases.
– Diseases induced by viruses (herpes, HPV, EBV,…).

The genomic instability appears in two different types:

– Chromosomal alterations in non-neoplastic precursor lesions and mutation of the P53 gene.
– Errors in DNA replication detected by microsatellite instability (deficiency in DNA mismatch repair mechanism).

We have observed (37, 69) such an instability in our studies with presence of chromosome copies number changes, chromosomal deletions, translocations, presence of endomitoses, premature centromeric disjunctions and presence of micronuclei (Figures 4 and 6A,B).

The loss of either essential genes (TSGs) or even entire chromosomes, explain the high invasive potential of the endometriotic cells. Genomic alterations (rearrangements) initiated for instance by telomere dysfunction can be a primary event that facilitate endometriosis initiation and spread (70).

## A Possible Pathogenic Role of Embryonic Duct Remnants in Female Fetal Reproductive Tract in Endometriosis and Ovarian Tumors

The theory of transformation of the vestigial tissue of Müllerian or Wolfian origin and the celomic metaplasia theory can explain the origin of distinct entities: for instance endometriotic lesions as well as development of particular types of ovarian neoplasms. In the same context, a recent study has proposed the fetal origin of endometriosis that could develop on the basis of altered migration of primitive endometrial tissue during embryogenesis (71). These authors assessed that the incidence of the dislocated embryonic structures in fetuses is similar to that of endometriosis occurring in the adult female population. In the same direction, relationship between endometriosis and malignancies arising in gonadal and extra gonadal endometrial implants become supported by several clinical pathologic and molecular investigations. These studies suggested that histogenetically, endometriosis represents an important site of origin of ovarian and other pelvic malignancies. It was described that such neoplasms are constituted of clear epithelial cells and tend to be detected in earlier stages, their prognosis being more favorable. In addition, embryonic duct remnants were often microscopically observed adjacent to ovarian tumors as well as close to pelvic endometriotic lesions suggesting a pathogenetic relationship (7).

**Figure 7:**
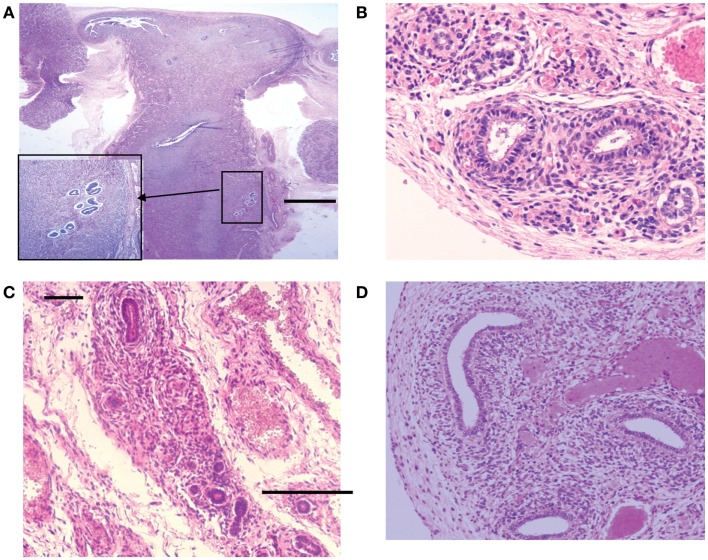
**Hematoxylin and eosin stained sections with areas of ectopic endometrial glands and embryonic ducts**. Histological appearance of ectopic glands and stroma observed (insert at higher magnification) in fetal uterine wall **(A)**. Presence of embryonic ducts located in the broad ligament **(B)**, under the fallopian tube serosa **(C)** and ducts located in the ovarian ligament **(D)**. Note presence of a stromal component surrounding the duct residues in a, b, c, and d. Scale bars, and 100 μm.

**Figure 8:**
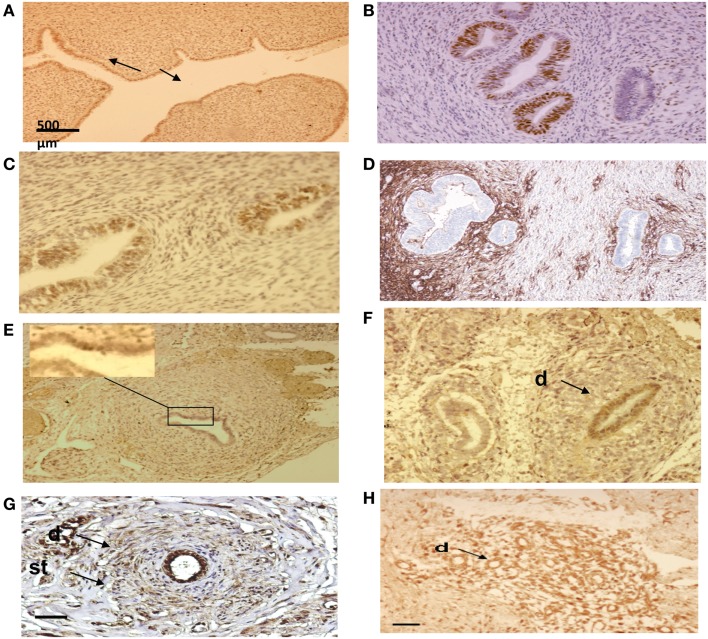
**Immuno-histo-chemical analysis of ectopic endometrial glands and embryonic ducts in fetal reproductive tract**. Immunolabeling with anti-PR antibody of uterine cavity wall lining cells [**(A)** arrows]. Immunostaining of the fetal ectopic glands located in the uterine myometrium with anti-PR **(B)**, anti ER-alpha **(C)**, and anti CD10 antibodies **(D)**. Expression of the PR in embryonic duct located in the ovarian ligament; insert at higher magnification **(E)**, expression of ER-α in a duct located under the fallopian tube serosa **(F)**, and expression of alpha-1-foetoprotein **(G)** and CD10 molecules **(H)**. D = duct; St = stromal layer. Scale bars; 500 μm in **(A)** and 100 μm in **(B–H)**.

In a previous study, we evaluated the incidence and the anatomical location of displaced endometrial tissue in the reproductive tract in seven female fetuses. Serial sectioning of the reproductive organs was realized followed by immuno-histochemical analysis of tissue areas containing ectopic glands and embryonic cell rests (Table 3; Figures 7 and 8).

**Table 3:** **Summary of the percentages of immunoreactive cells in embryonic ducts and the surrounding stroma present in various locations of the fetal reproductive tract**.

Gestational age (weeks)	EMA*		Cytokeratin 7*		ER*		PR*		Vimentin*		CD10*	
	D	St	D	St	D	St	D	St	D	St	D	St
18	32	–	2	–	10	–	19	11	35	4	2	55
19	52	–	–	–	12	–	21	6	26	9	–	38
20	38	–	–	3	18	3	13	8	34	13	–	42
21	43	–	–	–	13	–	19	4	22	20	–	39
22	46	–	–	–	23	–	31	13	25	6	–	44
32	0	0	0	–	0	0	0	0	0	0	0	0
36	53	1	11	1	16	1	44	9	53	32	4	64
Mean value**	264	1	13	4	92	4	147	51	195	84	6	282

D, embryonic duct; St, Stromal layer; *percentage of cells expressing the indicated marker; mean value** [represents a ratio of the number of counted cells (200) divided by the number of immunolabeled cells].

We have compared these results with those of two germ cells tumors, the young and adult forms using exclusively an immuno-histochemical analysis.

It was observed that the anatomical and the phenotypic features of the misallocated tissue components recall some pathological characteristics of both benign and malignant gynecological conditions (72).

## How and Why to Define the Evolutivity?

The actual classification used around the world is only anatomic, with adjunction recently of pain and deep lesions (76). But, we have observed with the immuno-histochemical analysis that the proliferation depend at first on the presence or not of hormonal receptors (PR; ER) and oncogenes.

In addition, in this study, the proliferation is shown with the lesions having lost their chromosomal stability. All these lesions have genomic abnormalities. Thus, when endometriotic cells cultured from these lesions (PR < 30% or PR-; ER-) are treated with progestins, an increase in the proliferation curve is observed (37) that is correlated with a clinical dissemination in the pelvis and possibly with the occurence of “metastasis” outside of the abdominal cavity.

**Figure 9:**
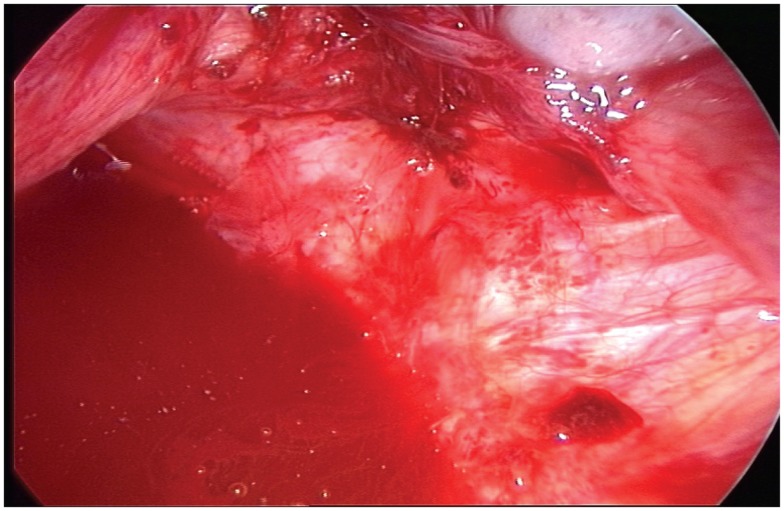
**Typical aspect of small red peritoneal excrescences in a context of extensive endometriosis**.

There are no correlations between the size of lesions and the severity of the disease (Figure 9). Consequently, all lesions removed by surgery must be tested by a phenotypic and genotypic analysis.

An immunohistochemical profile of the lesions (Endometriogram*) should be defined in a similar way as the procedure used to molecularly classify the breast cancer. Using this strategy, the medical treatment can be decided including the IVF technology in case of infertility.

Thus, larger prospective randomized studies should be carried on in the future with the main basic concept to introduce the most apporpriate algorithm for therapeutic decision.

## Conclusion and Perspectives

Substantial progress has been made in the last few years to understand better the etiologies, pathogenesis, and means to treat endometriosis. This field of investigation is supposed to make greater advances in the near future. From the collected molecular data, it is evident that one or several genes may be disrupted by mutations or loss of DNA sequences in this condition. Loss of some protein functions may effect the regulation of several emerging down stream pathways, including the EGFR-axis, E-cadherin dynamics, transcriptional regulation, cell cycle regulation (proliferation/apoptosis), and cell adhesion. Divergences from mature endometrial epithelium to endometriotic epithelium must involve abnormal gene expression, which may directly cause or reinforce the alterations caused by changes of one or several genes undergoing germinal alterations. Within this context, the treatment strategies of endometriosis centered mainly toward ovarian function suppression and indirect limitation of cell growth and activity of endometriosis remain insufficient (73, 74).

Future studies should concentrate on modifying and extending the use of the molecular biology techniques. Cytogenetic analysis with improved culture techniques of endometriotic tissue, capable of simulating the normal endometriotic environment, may help to prevent selective *in vitro* growth pressures. CGH and cDNA microarray studies has only been used to date on a limited number of primary endometriotic samples, yet has given us important informations (41, 75). Gene expression profiling, allelotyping, and FISH studies need to investigate the various types of endometriotic samples and more regions of the genome. As techniques are improving and examination of archival material is now possible it should be easier to correlate clinical data with the phenotypical features and genotype of primary lesions. This will certainly yield informations important for the early diagnosis and prognosis of patients.

Finally, the anatomical and the immuno-histochemical features of the ectopic organic structures identified in fetal female reproductive tract suggest that endometriotic as well as neoplastic disease in adult women may develop on the basis of misplaced endometrial glands and/or embryonic cell remnants.

## Author Contributions

All authors were equally involved in the design of the study, data acquisition, interpretation and analysis. Luca Gianaroli and Jean Marc Bernard Ayoubi were involved in samples preparation and analysis. Jean Gogusev was involved in manuscript preparation and performed the image and statistical data analysis.

## Conflict of Interest Statement

The authors declare that the research was conducted in the absence of any commercial or financial relationships that could be construed as a potential conflict of interest.
